# Marchiafava-Bignami Disease: Report of a Subacute Case

**DOI:** 10.7759/cureus.34692

**Published:** 2023-02-06

**Authors:** Ivo Castro, João Cardoso, Cristina Esteves, Adelaide Figueiredo

**Affiliations:** 1 Department of Internal Medicine, Hospital Distrital de Santarém, Santarém, PRT

**Keywords:** demyelinating neurological disorder, chronic alcoholism, magnetic resonance imaging, corpus callosum, marchiafava-bignami disease

## Abstract

Marchiafava-Bignami disease (MBD) is rare and often associated with chronic alcohol consumption; however, cases have been described in non-alcoholic patients with nutritional deficits. This disease manifests itself through an array of neurological signs and symptoms, from mild dysarthria or mild confusion to coma and death, and can present acutely, subacutely, or chronically, depending on their severity. The evolution of imaging technology makes magnetic resonance imaging (MRI) the gold standard for the diagnosis of this disease, although computed tomography (CT) scan is usually in the first line owing to its greater availability. The main feature for the diagnosis of MBD by brain MRI is the identification of areas of demyelination and necrosis of the corpus callosum. We report a 55-year-old male with subacute neurological deterioration whose MRI demonstrated atrophy and demyelination of the corpus callosum.

## Introduction

Marchiafava-Bignami disease (MBD) is a rare condition associated with chronic alcoholism, whose pathological characteristics involve demyelination or necrosis of the corpus callosum [1]. Sometimes the injuries even sprawl to the hemisphere white matter [2]. MBD affects mainly male patients, aged between 40 and 60 years, with history of chronic alcoholism and malnutrition. Clinically the diagnosis is difficult and relies upon imaging, particularly MRI, to demonstrate features of the disease [3].

This study aimed to describe the presentation and clinical approach to MBD with involvement of the corpus callosum, cortical and white matter.

## Case presentation

We present the case of a 55-year-old male with history of dyslipidemia, type 2 diabetes mellitus, hypertension, and alcoholic liver disease associated with significant daily abuse of alcohol for 35 years. He was admitted to hospital for anorexia, asthenia, a three-month history of worsening dysarthria, and rapidly declining mobility resulting in an inability to stand up and prostration. According to his family, he had stopped alcohol consumption one month prior to hospital admission. On examination, he was with normal body temperature, blood pressure, and heart rate. However he showed up drowsy, non-verbal, and carrying out simple orders; moreover, he showed ascites and gait instability. The patient did not have ophthalmoplegia or diplopia, despite the difficulty in assessing.

Laboratory investigations revealed mild leukocytosis with moderate neutrophilia, hyperglycemia (180 mg/dL), elevated aspartate aminotransferase levels (66 U/L), and ammonia (85 μg/dL) levels were normal. Other tests, such as international normalized ratio (INR), bilirubin and alanine aminotransferase (ALT) levels, c-reactive protein, renal function, folic acid, and cyanocobalamin levels were normal. A lumbar puncture was performed, and the cerebrospinal fluid analysis ruled out inflammation or infection. The ascitic fluid analysis did not show signs of infection and the serum-ascites albumin gradient was compatible with alcoholic chronic liver disease.

**Figure 1:**
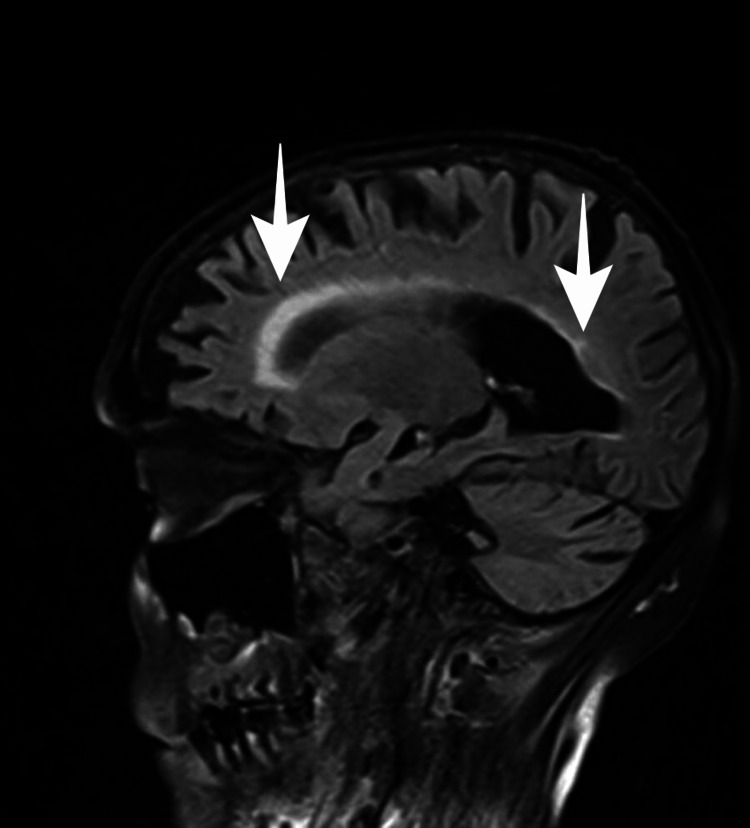
MRI sagittal T2 FLAIR images showing hyperintense lesions in the corpus callosum. FLAIR: fluid-attenuated inversion recovery

**Figure 2:**
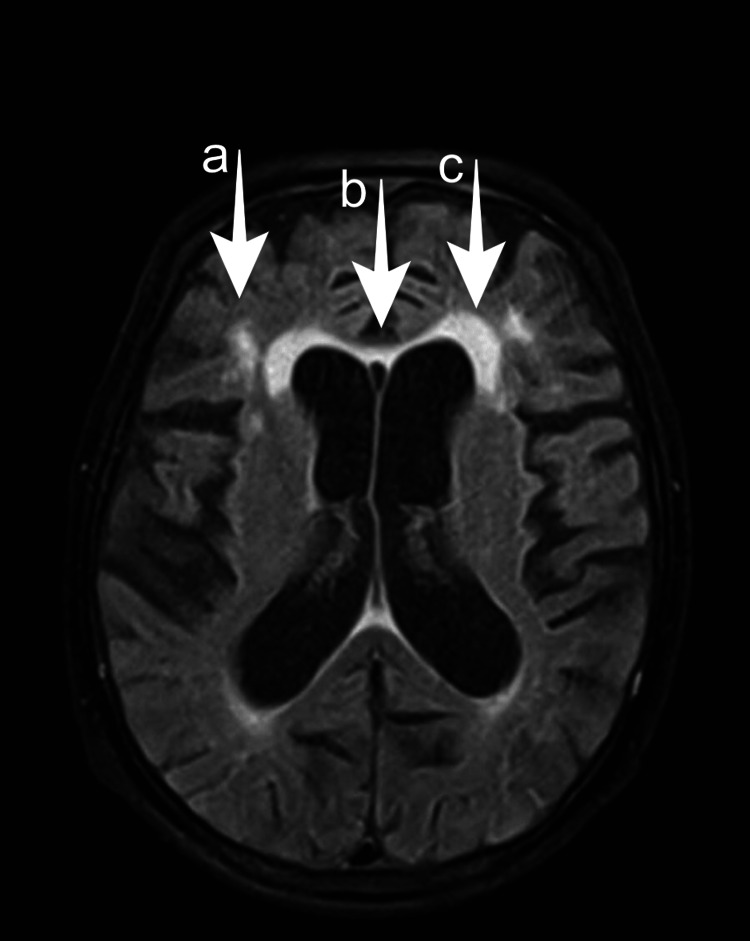
MRI axial T2 FLAIR images showing hyperintense lesions in (a) deep periventricular matter, (b) corpus callosum, and (c) deep white matter. FLAIR: fluid-attenuated inversion recovery

Two computed tomography (CT) scans of the brain, taken 20 days apart, were unremarkable. MRI of the brain revealed atrophy and demyelination of the corpus callosum with a hyperintensity of the corpus callosum observed in long Repetition Time (TR) sequences, with anterior and posterior predominance, specifically in fluid-attenuated inversion recovery (FLAIR), as well as the atrophic phenomenon of this structure (Figures 1, 2). Additionally, MRI of the brain also revealed important cortical-subcortical atrophy, marked white matter hypersignal at the periventricular level and discrete hypersignal foci on the same sequences at the periventricular level, white matter of the corona radiata, and semi-oval centers.

Therefore, a diagnosis of Marchiafava-Bignami was made on the basis of clinical information, physical examination, and imaging findings. The patient was hospitalized for 33 days, receiving thiamine, vitamin B complex, and folic acid. Although patient's consciousness level improved, along with a slight regression of dysarthria and mobility, he then developed severe pneumonia that evolved into septic shock and a comatose state. The patient died from hospital-acquired pneumonia.

## Discussion

MBD was initially described by two Italian pathologists after observing the degeneration of the corpus callosum in the autopsy of three patients. The patients had consumed excessive amounts of cheap red wine and presented with seizures and coma [3-5].

Although the pathophysiology is unclear, MBD is an uncommon degeneration of the corpus callosum, resulting from demyelination, necrosis, and occasionally with hemorrhages [1,4]. Despite mainly occurring in patients with chronic alcohol consumption, MBD has been diagnosed in patients with malnutrition or frequent vomiting were also identified. Both patients with alcoholism and those diagnosed with malnutrition are linked by nutritional deficits, namely thiamine, vitamin B complex, and folate. However, after supplementation, not all patients improved [4].

The clinical presentation of MBD is variable, with diverse neurological symptoms and signs, and can be described as acute, subacute, or chronic [4]. The acute presentation has a sudden onset with altered consciousness, seizures, coma, and rapid evolution to death. The subacute presentation is characterized by behavioral changes, memory deficit, mental confusion, and an altered gait. The least common of presentations is the chronic one, which manifests as progressive dementia with insidious evolution over years [1,2,4].

The gold standard for MBD diagnosis is brain MRI [4]. CT imaging is not sensitive enough to identify early lesions. The characteristic findings on MRI are symmetrical lesions of the corpus callosum, but lesions can also be identified in the cortex, white matter, middle cerebellar peduncles, and internal capsules [6,7].

In the differential diagnosis of MBD, other alcohol-associated diseases should be considered. These include neoplastic conditions, multiple sclerosis, epilepsy, and Wernicke's encephalopathy. Wernicke's encephalopathy usually manifests with ataxia, ocular dysfunction, namely nystagmus and ophthalmoparesis, disorientation and treatment with thiamine leads to a recovery in a short period, as opposed to MBD whose recovery is slow [4].

There is no defined treatment; however, early diagnosis and treatment with thiamine, folic acid, and B complex vitamins can accelerate and enhance recovery. The use of corticosteroids aims to reduce inflammation and stabilize the blood-brain barrier [4].

The mortality of MBD is high, and the acute form often progresses quickly to death. Patients who survive are left with severe neurological deficits [4]. Some patients with milder manifestations occasionally have partial or complete recovery [3].

Patients with early diagnosis, circumscribed lesions on MRI, and who are quickly treated have a better prognosis [8]. MBD of alcoholic etiology is associated with a worse prognosis, with infectious complications being the main causes of death [9].

## Conclusions

MBD is a rare neurological disease, usually associated with heavy and prolonged alcohol consumption and high mortality. Although widely available, CT has a low level of sensitivity and MRI is the imaging study of choice. The MRI identification of areas of corpus callosum degeneration with demyelination and necrosis are important for the diagnosis. There is currently no defined treatment; however, nutritional supplementation, specifically with B complex vitamins, seems to aid clinical improvement. The best chance of clinical improvement is through early diagnosis, making clinical suspicion a crucial factor.
